# Transarterial Chemoembolization Treatment Paradigms for Hepatocellular Carcinoma

**DOI:** 10.3390/cancers16132430

**Published:** 2024-07-01

**Authors:** Elliott L. Fite, Mina S. Makary

**Affiliations:** 1 College of Medicine, The Ohio State University, Columbus, OH 43210, USA; elliott.fite@osumc.edu; 2 Department of Radiology, The Ohio State University Medical Center, Columbus, OH 43210, USA

**Keywords:** transarterial chemoembolization, hepatocellular carcinoma, liver cancers

## Abstract

**Simple Summary:**

Hepatocellular carcinoma (HCC) is the most common primary liver cancer, comprising 90% of liver cancer cases worldwide, with a five-year survival rate of less than 20%. Standard treatments for early-stage HCC include surgical resection and liver transplantation, but many patients are diagnosed at later stages and are ineligible for these interventions. For such patients, transarterial chemoembolization (TACE) is an alternative treatment that delivers chemotherapy directly to the tumor and blocks its blood supply. TACE can be used to help patients maintain eligibility for transplants, improve liver function, and alleviate symptoms. It is particularly effective for intermediate-stage HCC but also shows promise in the early and advanced stages when combined with other therapies. While generally safe, TACE can cause post-embolization syndrome (PES) and other complications in a minority of cases. The efficacy of TACE is continually being improved through advancements in techniques and the integration of novel systemic therapies and artificial intelligence for better patient selection and outcomes.

**Abstract:**

Hepatocellular carcinoma (HCC) accounts for 90% of liver cancer cases worldwide and is currently the most quickly increasing cause of cancer-related deaths in the United States. The 5-year survival rate for primary liver cancer is estimated to be below 20%, and HCC mortality is expected to increase by 41% by 2040. Currently, surgical resection is the first-line approach to definitive treatment of early-stage HCC. However, the majority of patients present with late-stage, unresectable disease due to the asymptomatic nature of early HCC. For patients who present with unresectable HCC, locoregional therapies such as transarterial chemoembolization (TACE) represent an alternative approach to HCC treatment. TACE is a minimally invasive, catheter-based technique that allows for targeted delivery of chemotherapy to tumor sites while occluding tumor-feeding blood vessels. In appropriately selected patients, outcomes for TACE therapy have been shown to be more favorable than supportive care or conservative management. The increasing incidence and mortality of HCC, in addition to the late-stage presentation of most HCC patients, demonstrates the need to expand the role of locoregional therapies in the treatment of HCC. TACE represents an appealing approach to HCC management, including disease control, palliation, and potentially curative-intent strategies. In this review, we will describe the current utility of TACE in the treatment of HCC, characterize the outcomes of patients treated with TACE across different HCC stages, and outline future applications of TACE in the treatment paradigm.

## 1. Introduction

### Hepatocellular Carcinoma

Hepatocellular carcinoma (HCC) is the most common primary liver malignancy, accounting for 90% of liver cancer cases worldwide [1]. HCC-associated mortality is high, with an estimated 5-year survival rate of less than 20% [1]. Surgical resection and liver transplantation remain the standard first-line treatments for early-stage HCC. Patients undergoing resection for early-stage HCC have an overall 5-year survival of 50–70% [2,3], with >60% of patients having recurrent HCC 5 years post-resection [4,5]. Liver transplantation has similar outcomes in patients with early-stage HCC, with a 5-year overall survival rate of >70% [6] and a recurrence rate of 6–15% [7]. However, due to the asymptomatic nature of early-stage HCC, patients often present with late-stage disease and are therefore not candidates for surgical treatment. In a study of 8000 patients with HCC, less than 10% fulfilled the pre-operative criteria for resection [8]. For patients who do not qualify for surgical treatment of HCC, locoregional therapies like transarterial chemoembolization (TACE) represent an alternative approach to therapy.

TACE is a minimally invasive, catheter-based approach that involves targeted delivery of chemotherapeutic agents while occluding tumor-feeding blood vessels using embolic agents [9]. In appropriately selected patients, outcomes for TACE therapy have been shown to be more favorable than supportive care or conservative management alone [10]. TACE is a versatile treatment modality and can be used to downstage HCC patients for transplant eligibility, enhance liver function following resection, palliate symptoms, and in some instances, cure disease [11].

## 2. Staging in Hepatocellular Carcinoma

The Child–Pugh score is a widely used clinical tool to assess the severity of liver disease, including patients with cirrhosis and HCC [12]. Clinically, the Child–Pugh score is used to determine the prognosis and appropriate treatment strategies for patients with liver disease based on five clinical and laboratory parameters: encephalopathy, ascites, bilirubin, albumin, and prothrombin time [13]. Each parameter is assigned a score from 1 to 3, and the total score is then used to classify patients into one of three classes: A, B, or C. A Child–Pugh Class A classification represents well-compensated disease and corresponds to the best prognosis with the longest expected survival. Child–Pugh Class B signifies significant functional compromise; these patients have moderate liver dysfunction with limited treatment options. Patients with severe liver dysfunction are classified as Child–Pugh Class C, representing a decompensated state. These patients have the worst prognosis, and treatment efforts are generally focused on supportive care, as aggressive interventions are often not feasible. In the management of hepatocellular carcinoma, the Child–Pugh score is an integral tool for assessing treatment eligibility, providing prognostic information about overall survival and treatment outcomes, and supporting clinical decision making via its integration in staging systems like the Barcelona Clinic Liver Cancer (BCLC) staging system.

The BCLC staging system is a comprehensive classification system used to stage hepatocellular carcinoma and guide treatment decisions. It integrates various prognostic factors, including tumor characteristics, liver function, and patient performance status to stratify patients into different stages based on disease severity (Table 1) [14]. The BCLC system broadly incorporates the following: (1) tumor characteristics, defined by the size and number of nodules as well as vascular invasion of the tumor; (2) liver function using the Child–Pugh scoring system; and (3) performance status using the Eastern Cooperative Oncology Group (ECOG) system [15]. The BCLC system is divided into five categories ranging from very early-stage HCC (BCLC stage 0) to terminal HCC (BCLC stage D) (Table 1). Studies have shown TACE to be an effective treatment modality across all BCLC stages, and its role in the management of HCC continues to expand with the development of new technologies and the refinement of existing strategies.

## 3. Patient Selection for TACE Therapy

TACE is most appropriate for patients with intermediate-stage HCC (BCLC Class B, Child–Pugh B or better) who are ineligible for liver transplant or surgical resection (Table 1) [16]. Patients with very early-stage (BCLC 0) and early-stage (BCLC A) HCC with no underlying liver disease (Child–Pugh Class A) should undergo surgical resection as a first-line treatment approach (Table 1). However, TACE may be indicated for patients who are ineligible for surgical resection or for those patients who would benefit from downstaging to maintain transplant eligibility [17]. Early-stage HCC patients may be ineligible for surgical resection for a multitude of reasons, including poor liver function (Child–Pugh Class C), the presence of comorbidities like cardiovascular or pulmonary disease, tumor location, the presence of multifocal disease, or poor overall health [18]. For these patients, TACE can represent a safe and effective alternative for locoregional control of HCC [17].

TACE can also be used as a means to maintain transplant eligibility through downstaging patients with intermediate-stage HCC. Downstaging aims to reduce tumor size and numbers to meet criteria for liver transplants, such as the Milan criteria (a single tumor of <5 cm or up to three tumors that are each < 3 cm without vascular invasion) [19] and the UCSF criteria (a single tumor of <6.5 cm or up to three tumors with the largest lesion being <4.5 cm and having a total tumor diameter of <8 cm) [20]. Studies have shown that TACE can effectively downstage tumors in a significant proportion of patients [21], and patients who are successfully downstaged to meet transplant criteria have comparable post-transplant survival rates to those who initially met the criteria without downstaging [22].

TACE is a widely used and effective treatment modality for HCC. However, several contraindications must be considered to ensure patient safety and treatment efficacy. Absolute contraindications for TACE include decompensated cirrhosis (Child–Pugh B or higher), reduced portal vein flow, creatinine clearance < 30 mL/min, and bi-lobe tumor involvement [23]. Relative contraindications for TACE treatment include severe comorbidities, high tumor burden, and elevated liver function markers [14]. While TACE is a valuable treatment method for HCC, it is essential that each patient undergo a thorough evaluation prior to treatment, including liver function tests, imaging studies, and assessment of overall health status, to minimize risks and optimize treatment outcomes.

## 4. TACE Technique

**Figure 1:**
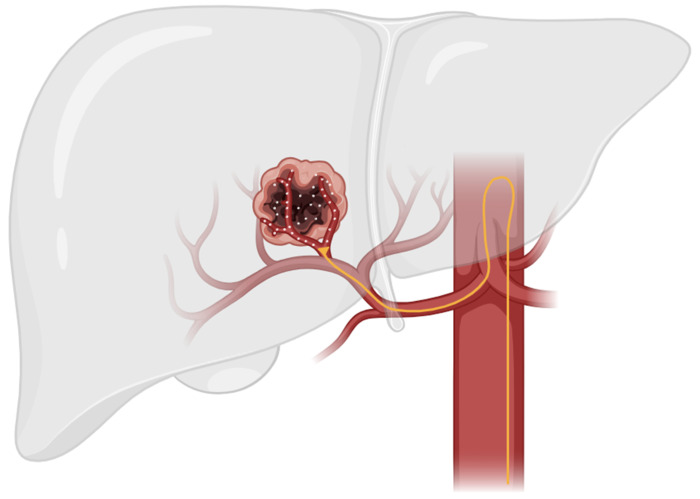
This figure illustrates the general technique used in TACE therapy. In TACE, a catheter is guided through the hepatic artery to the site of the tumor. Localized therapeutic agents are then delivered directly to tumor parenchyma while sparing healthy liver tissue.

TACE is a minimally invasive approach to HCC treatment that utilizes the anatomic difference in blood supply between normal liver parenchyma and hepatocellular tumors. These tumors have an increased metabolism compared to normal liver parenchyma and, therefore, must promote angiogenesis to increase blood flow in order to meet the increased metabolic demand. The normal liver parenchymal is primarily supplied by the portal venous system, while hepatocellular tumors are primarily supplied by the hepatic arterial system [24]. This anatomic difference enables targeted delivery of therapeutic agents via TACE through the hepatic arterial supply while sparing healthy liver tissue (Figure 1) [10].

Multiple approaches to TACE therapy exist based on the anatomic location of drug delivery, including lobar, selective, and super-selective. In lobar administration, therapeutic agents are delivered to the lobar branch of the hepatic artery, resulting in drug delivery to the entire lobe containing HCC. In contrast, selective and super-selective TACE techniques improve the precision of chemotherapy delivery by administering drugs to a segmental artery (selective) or sub-segmental hepatic artery (super-selective) directly [25]. When compared to lobar TACE, selective/super-selective TACE have demonstrated increased levels of tumor necrosis (75.1% versus 52.8%, *p* = 0.002) [26]. Overall, selective and super-selective TACE showed complete response rates between 40% and 50% [27] and a 5-year overall survival between 20% and 30% [28].

In addition to variations in anatomic location, there are also two main techniques used in TACE therapy for drug delivery: conventional TACE (c-TACE) and drug-eluting bead TACE (DEB-TACE). In c-TACE, a lipiodolized chemotherapeutic agent is delivered to the tumor via a catheter advanced into the hepatic artery, followed by an embolic agent. This approach increases the chemotherapeutic concentration in the tumor while minimizing pharmacologic washout [29]. Alternatively, DEB-TACE utilizes drug-eluting beads to deliver chemotherapeutic agents in a sustained fashion, improving standardization and potentially decreasing hepatotoxicity compared to c-TACE [30]. Both DEB-TACE and c-TACE use chemotherapeutic agents to embolize the arteries supplying a tumor, creating an ischemic state that promotes tumor necrosis [31]. Common chemotherapeutic agents used in c-TACE and DEB-TACE include doxorubicin, cisplatin, and mitomycin C [9]. Traditionally, these three agents were given together; however, recent trends in clinical practice have increasingly seen doxorubicin used as a monotherapy [32]. Guidelines for the choice and dosing of chemotherapeutic agents used in TACE therapy are currently lacking. The currently used dose range for doxorubicin is 10–100 mg, while cisplatin is 10–100 mg, and mitomycin C is 2–30 mg [33]. Dosages for the chemotherapeutic agents used in TACE can be based on body surface area, liver function, or weight, or they can be empirically determined [32]. Even without a standardized dosing regimen, DEB-TACE and c-TACE have been shown to provide superior therapeutic targeting when compared to systemic chemotherapy [34].

All approaches (lobar, selective, and super-selective) and techniques (c-TACE vs. DEB-TACE) used in TACE therapy have been demonstrated to be safe, but there are several known risks. Up to 80% of patients undergoing TACE therapy may experience post-embolization syndrome (PES) [10]. PES consists of a fever post-embolization in the absence of infection, as well as abdominal pain, nausea, or vomiting. While PES is common among patients following TACE procedures, most cases of PES resolve within 72 h, and very few cases of PES progress to serious clinical consequences [35]. Rare but serious complications associated with TACE occur in approximately 5% of cases and include abscess, acute cholecystitis, iatrogenic dissection, and acute hepatic failure [36].

## 5. TACE Outcomes

### 5.1. Outcomes for TACE in Early-Stage HCC

TACE has primarily been studied in intermediate-stage HCC; however, a small prospective study of selective TACE in early HCC suggests that TACE may be an effective treatment approach for early-stage HCC patients [37]. Bargellini et al. conducted a prospective cohort study of TACE therapy in very early- and early-stage HCC patients deemed clinically unfit for liver transplantation [37]. A total of 67 patients with BCLC stage 0 or A HCC were included in the study and were treated with TACE via selective catheterization of the hepatic segmental arteries [37]. At 1 month, 67.2% (*n* = 45) of patients experienced a complete response following TACE therapy while 29.8% (*n* = 20) experienced a partial response. Overall survival rates were 90.9%, 86.1%, and 80.5% at 1, 2, and 3 years, respectively (Table 2) [37]. A total of 12 patients (17.9%) were observed to have radiologic disease progression with a mean expected time of progression of 26.5 months [37].

ALT and bilirubin values were significantly increased from preprocedural values; however, both the mean ALT and bilirubin levels decreased by the time of discharge. Liver failure occurred in 3% (*n* = 2) of patients, both with a Child–Pugh score of Class B [37]. Post-embolization syndrome was observed in 58.2% of patients. This study demonstrated the efficacy of TACE in achieving favorable early tumor response rates without increasing the rate of major complications in early-stage HCC. Similarly, multiple studies show that the patients most likely to benefit from TACE are those with persevered liver function and fewer lesions, suggesting that TACE may represent a safe and effective approach to the management of very early- and early-stage HCC in patients [25].

TACE after resection may also benefit early-stage HCC patients with microvascular involvement [44]. Multiple meta-analyses have shown that adjuvant TACE improved overall survival and disease-free survival in patients with resected HCC [45]. Overall, adjuvant TACE following HCC resection had a 1-year survival rate of 28–82% and a 3-year survival rate of 32–43.9% [46].

The GIDEON study used observational data to show that patients given TACE concomitantly with sorafenib achieved better survival outcomes (21.6 months) compared with patients treated with sorafenib alone, a result that was consistent in early-stage (BCLC A) HCC patients [38]. While observational in nature, the results of this study demonstrate the versatile applicability of TACE therapy in the treatment of early-stage HCC. However, TACE is not currently indicated as a first-line treatment for early-stage HCC according to the 2022 BCLC guidelines [15]. The clinical utility of the available data on TACE treatment for early-stage HCC is limited due to the retrospective/observational nature of the studies or the small sample sizes of prospective studies. Nevertheless, the promising data that are available supporting the effectiveness of TACE in treating early-stage HCC patients with unresectable disease, as well as the development of new approaches such as concomitant TACE and sorafenib treatments, highlight the potential for TACE therapy in the management of early-stage HCC in patients.

### 5.2. Outcomes for TACE in Intermediate-Stage HCC

TACE is the first-line treatment option for intermediate-stage HCC patients (BCLC B, Child–Pugh B or better) with preserved liver function, well-defined nodules, and preserved portal flow (Table 1) [18]. If patients are carefully selected based on these parameters, there is ample evidence suggesting that TACE can improve survival. In a review of 41 BCLC stage B HCC patients, Burrel et al. reported 1-, 3-, 4-, and 5-year survival rates of 88.2%, 64.4%, 47.3%, and 39.4%, respectively (Table 2) [40]. Additionally, the median overall survival of BCLC stage B patients was 47.7 months (95% CI: 32.7–62.7) (Table 2) [40]. Taken together, the results of this study support the use of DEB-TACE in appropriately selected intermediate-stage HCC patients.

Combining TACE with systemic sorafenib chemotherapy has also been investigated in intermediate-stage HCC. Sorafenib inhibits tumor growth and progression by blocking the Raf pathway and also inhibits angiogenesis by blocking the VEGF pathway in endothelial cells [47]. Ischemia following TACE therapy can produce neoplastic angiogenic growth factors, an effect that can potentially be mitigated via the anti-angiogenic effects of sorafenib [9]. The GIDEON study used global observational data to evaluate the survival outcomes of HCC patients treated with sorafenib with or without concomitant TACE. Intermediate-stage (BCLC B) HCC patients treated with concomitant TACE and sorafenib had a median overall survival of 27.0 months compared to 14.2 months for patients treated with sorafenib alone (Table 2) [38]. No significant difference in the incidence of adverse events was observed with concomitant sorafenib and TACE compared to sorafenib alone (Table 2) [38]. The results of the GIDEON study suggest that TACE may be effective in treating intermediate-stage HCC given concomitantly with systemic sorafenib. However, the observational nature of the data limits the clinical utility of the study.

The SPACE trial addressed this shortcoming by prospectively randomizing 307 intermediate-stage (BCLC B) HCC patients in a 1:1 ratio to either DEB-TACE with sorafenib or a placebo [41]. While the results of the SPACE trial showed that DEB-TACE plus sorafenib was safe and feasible, it did not improve the time to progression in a clinically meaningful manner (Table 2). More recently, the Phase II TACTICS trial randomized 156 patients with unresectable HCC to receive either TACE alone (*n* = 76) or sorafenib in combination with TACE (*n* = 80) [42]. The TACTICS study found a progression-free survival (PFS) of 25.2 months in patients treated with TACE and sorafenib compared to 13.5 months in patients treated with TACE alone (Table 2) [42]. Additionally, the time to progression (TTP) was significantly improved with TACE combined with sorafenib therapy, and no unexpected toxicities were observed (Table 2) [42]. The results of the SPACE and TACTICS trials demonstrate that TACE can be used as a safe and effective combination with sorafenib treatment in the management of intermediate-stage HCC.

### 5.3. Outcomes for TACE in Advanced HCC

For patients with advanced HCC who may be unable to tolerate the side effects of systemic therapy, TACE may be considered an alternative approach to treatment. TACE has been shown to have superior outcomes to conservative therapy alone in treating patients with advanced HCC [48]. Advanced-stage HCC (BCLC stage C) patients are typically treated using systemic therapy in the form of sorafenib, Lenvatinib, or more recently, atezolizumab and bevacizumab [49,50]. TACE may be considered alone or in combination with systemic therapy when local disease control is needed in advanced HCC. The GIDEON study found that patients with advanced HCC (BCLC stage C) treated with concomitant TACE and sorafenib therapy had a median overall survival of 15.5 months compared 8.3 months with sorafenib alone (Table 2) [38]. Similarly, Zhang et al. conducted a meta-analysis evaluating the safety and efficacy of TACE therapy combined with sorafenib in advanced-stage HCC patients [43]. The results showed that TACE combined with sorafenib therapy significantly improved overall survival (HR = 0.65; 95% CI: 0.47–0.89, *P* = 0.007) and time to progression (HR = 0.68; 95% CI: 0.52–0.87, *P* = 0.003) (Table 2) [43]. TACE is also currently being evaluated in combination with Lenvatinib and pembrolizumab for advanced non-metastatic disease [51].

## 6. Future Directions

The future directions of TACE in the treatment of HCC are focused on enhancing its efficacy through integration with novel immunotherapies, developing new technologies for administering TACE, and validating artificial intelligence (AI) models to estimate responses to TACE treatment.

As previously discussed, current research trends are extensively studying the use of TACE in combination with target therapies like sorafenib and Lenvatinib. These combinations aim to improve overall survival and progression-free survival by targeting the primary tumor and any potential micro-metastases [52]. Novel research on systemic immunotherapies in the treatment of intermediate- and advanced-stage HCC continues to show promise. In the IMBRAVE150 trial, patients with unresectable HCC treated with atezolizumab combined with bevacizumab showed better overall survival and improved safety, specifically lower rates of liver toxicity, compared to systemic sorafenib alone [53]. As previously discussed, concurrent TACE and sorafenib can improve overall survival in HCC treatments [38]. The efficacy of concurrent TACE and sorafenib therapy, in addition to the improved survival and safety of immunotherapies compared to sorafenib, suggests that a combination of TACE and immunotherapies may have a synergistic effect. Multiple prospective trials are currently investigating this combination. The Phase 3 LEAP 012 trial will evaluate the clinical benefit of combining Lenvatinib and pembrolizumab with TACE therapy in patients with intermediate-stage HCC [54]. Additional studies evaluating immunotherapies and TACE treatment include the IMMUTACE (NCT03572582) and EMERALD-1 (NCT03778957) trials [55]. The results of these multi-centered trials have the potential to alter treatment paradigms on the use of TACE to treat HCC. Continued research to determine the optimal sequencing and combination of TACE with systemic therapies will help develop comprehensive treatment plans and may expand the role of TACE therapy in the treatment of HCC [56].

Along with advancements in combinations of TACE and systemic therapy, recent developments in TACE techniques have also shown potential to improve outcomes in HCC. Specifically, preliminary results suggest that balloon-occluded TACE (B-TACE) may be superior to c-TACE. B-TACE involves the occlusion of feeding arteries by a micro-balloon catheter with the infusion chemotherapeutic agent lipiodol, allowing for dense lipiodol accumulation in the target nodules [57]. Multiple retrospective studies have demonstrated B-TACE to have an improved therapeutic effect when compared to c-TACE [58,59]. However, randomized controlled trials evaluating the efficacy of B-TACE compared to other TACE methods are lacking. Further research on B-TACE is needed in order to change clinical decision making.

AI models to evaluate response to initial TACE treatment and estimate 1-year survival following TACE are currently being trained. Peng et al. used computed tomography (CT) scans of patients with HCC to train and validate an AI model to predict response rates to TACE [60]. Patients were selected based on the following criteria: (1) radiologically or pathologically proven HCC; (2) received initial TACE treatment; (3) availability of hepatic arterial CT imaging 7 days before treatment and 30 days after treatment; and (4) BCLC stage B HCC [60]. Across three different training sets, they reported accuracies of 84.3%, 85.1%, and 82.8%, respectively, for predicting response rates to TACE therapy [60]. Mähringer-Kunz et al. developed a prediction model for 1-year survival following TACE for HCC patients [61]. This study included only TACE-naïve patients with HCC confined to the liver and who underwent at least two TACE treatments [61]. Overall, their model had a positive predictive value of 87.5% and a negative predictive value of 68.0%, with a sensitivity and specificity of 77.8% and 81.0%, respectively [61]. The work of Peng et al. and Mähringer-Kunz et al. demonstrates the potential of AI models as predictive tools that may be able to help clinicians better screen patients with HCC who can benefit from TACE treatment [60,61].

In addition to refining the use of TACE in primary HCC, current research efforts are also exploring an expanded role of TACE therapy in the treatment of metastatic disease of the liver. Specifically, TACE has shown promising tumor response rates and overall survival (OS) compared to systemic chemotherapy in colorectal cancer with liver metastasis (CRLM), breast cancer with liver metastasis (BCLM), uveal melanoma with liver metastasis (UMLM), and intrahepatic cholangiocarcinoma (IHC) [62]. These promising results, paired with the recent advances in improving the safety and efficacy of TACE therapy discussed in this review, suggest that further research into the clinical utility of TACE in the management of metastatic disease of the liver is warranted.

Finally, the need for further standardization and optimization of current TACE protocols is clear. The lack of a standardized protocol in TACE therapy is consistently cited as a key limitation in translating promising data on TACE outcomes into clinical practice [38]. Developing and updating comprehensive clinical guidelines for TACE procedures will ensure consistent and effective treatment across different healthcare settings, which in turn will facilitate research to optimize TACE practices.

## 7. Conclusions

TACE stands as a crucial treatment modality in the management of HCC, particularly for patients who are ineligible for surgical resection or liver transplantation. TACE is widely effective across various stages of HCC and is particularly useful as a first-line treatment for intermediate-stage HCC or as a valuable adjunct in the early and advanced stages.

The efficacy of TACE is supported by numerous studies demonstrating significant improvements in overall survival and progression-free survival, especially when combined with systemic therapies, such as sorafenib. Recent advancements in TACE techniques, such as B-TACE, may also contribute to improved therapeutic effects and patient outcomes. Emerging technologies, including the use of artificial intelligence to predict treatment response and survival rates, offer exciting possibilities for enhancing patient selection and personalizing TACE therapy. The continued exploration of TACE in combination with novel systemic treatments, particularly immunotherapies, holds the promise of further expanding its role in the multidisciplinary management of HCC.

However, the application of TACE is not without limitations. The procedure carries risks, including PES and other rare but serious complications. Additionally, the lack of standardized TACE protocols remains a significant barrier to optimizing treatment outcomes. Future research should focus on refining patient selection criteria, minimizing adverse effects, and establishing standardized protocols to ensure consistent and effective TACE practices across healthcare settings.

In conclusion, TACE represents a versatile and effective treatment option for HCC, offering substantial benefits in terms of tumor control and patient survival. Ongoing research and technological advancements are poised to further improve the efficacy and safety of TACE, solidifying its place as a cornerstone in the fight against HCC.

## Figures and Tables

**Table 1:** Barcelona Clinic Liver Cancer (BCLC) staging and treatment recommendations for HCC. This table describes the BCLC classifications of HCC as well as the current first-line treatment guidelines for each BCLC class [15]. PS: performance status.

BCLC Classification	Stage	Definition	BCLC Treatment Guideline
0		Single nodule	
	Very early stage	≤2 cm	Resection or liver transplant
		PS = 0	
A	Early stage	Single or ≤3 nodules	Resection or liver transplant
		Each nodule ≤ 3 cm	
B		Multinodular	TACE
	Intermediate stage	Preserved liver function PS = 0	
C		Portal invasion and/or extrahepatic spread	Systemic treatment
	Advanced stage	Preserved liver function PS = 1–2	
D		Any tumor burden	Best supportive care
	Terminal stage	End-stage liver function PS = 3–4	

**Table 2:** Outcomes after TACE therapy across HCC stages. This table outlines overall survival, progression-free survival, and adverse events reported in studies evaluating TACE treatment in early-, intermediate-, and advanced-stage HCC. BCLC: Barcelona Clinic Liver Cancer; TACE: transarterial chemo embolization; DEB-TACE: drug-eluting bead TACE; OS: overall survival; TTP: time to progression; HR: hazard ratio; PES: post-embolization syndrome.

Stage of HCC	Study	TACE Method	Overall Survival	Progression-Free Survival	Adverse Events
Early (BCLC 0-A)	Bargellini et al. [37]	Selective	1 year: 90.9% 2 years: 86.1% 3 years: 80.5%	Disease progression observed in 17.9% of patients, with a mean expected time of 26.5 months	Increased ALT and bilirubin 58.2% PES
	GIDEON [38]	Concomitant TACE and sorafenib	Median OS: 21.4 months	Not reported	No unexpected toxicity
	Chen et al. [39]	Adjuvant TACE following HCC resection	1 year: 28–82% 3 years: 32–43.9%	Not reported	Not reported
Intermediate (BCLC B)	Burrel et al. [40]	DEB-TACE	1 year: 88.2% 3 years: 64.4% 4 years: 47.4% 5 years: 39.4%	Not reported	Not reported
	GIDEON [38]	Concomitant TACE and sorafenib	Median OS: 27 months	Not reported	No unexpected toxicity
	SPACE [41]	DEB-TACE +/− sorafenib	Not reported	Median TTP: 169 days	Safe and feasible
	TACTICS [42]	TACE +/− sorafenib	Not reported	PFS: 25.2 months TTP: 24.1 months	No unexpected toxicity
Advanced (BCLC C)	GIDEON [38]	Concomitant TACE and sorafenib	Median OS: 15.5 months	Not reported	Not reported
	Zhang et al. [43]	TACE + sorafenib vs. TACE alone	TACE + sorafenib improved OS: HR = 0.65	TACE + sorafenib improved TTP: HR = 0.68	Increased incidence of grade III/IV adverse reactions compared to TACE alone
